# Processing Self-Related Information Under Non-attentional Conditions Revealed by Visual MMN

**DOI:** 10.3389/fnhum.2022.782496

**Published:** 2022-04-06

**Authors:** Sizhe Cheng, Xinhong Li, Qingchen Zhan, Yapei Wang, Yaning Guo, Wei Huang, Yang Cao, Tingwei Feng, Hui Wang, Shengjun Wu, Fei An, Xiuchao Wang, Lun Zhao, Xufeng Liu

**Affiliations:** ^1^Department of Military Medical Psychology, Air Force Medical University, Xi’an, China; ^2^Department of General Medicine, Tangdu Hospital, Xi’an, China; ^3^Department of Psychiatry and Psychology, 923 Hospital of Joint Logistic Support Force of Chinese People’s Liberation Army, Nanning, China; ^4^School of Education Science, Liaocheng University, Liaocheng, China

**Keywords:** self-related information, preattentional processing, change detection, ERPs, vMMN

## Abstract

Mismatch negativity (MMN) of event-related potentials (ERPs) is a biomarker reflecting the preattentional change detection under non-attentional conditions. This study was performed to explore whether high self-related information could elicit MMN in the visual channel, indicating the automatic processing of self-related information at the preattentional stage. Thirty-five participants were recruited and asked to list 25 city names including the birthplace. According to the difference of relevance reported from the participants, we divided names of the different cities into high (birthplace as deviants), medium (Xi’an, where participants’ university is located, as deviants), and low (totally unrelated cities as standard stimuli) self-related information. Visual MMN (vMMN) was elicited by high self-related information but not by medium self-related information, with an occipital–temporal scalp distribution, indicating that, under non-attentional condition, high self-related information can be effectively processed automatically in the preattentional stage compared with low self-related information. These data provided new electrophysiological evidence for self-related information processing.

## Introduction

Self-related information (SRI) refers to information that is related to oneself but has nothing to do with others, that has individual meaning and social value, and that shows processing characteristics different from others’ information (Wang, 2014). Individuals have a strong ability to recognize their own physiological and psychological information, such as recognizing their own face, voice, name, and autobiographical memory according to different SRI clues (Gallup, 1985; Gillihan and Farah, 2005; Gallup et al., 2014).

The process of attention captured by SRI might be unconscious, uncontrollable, and non-attentional automatic processing. According to Northoff’s (2016) research, SRI processing was the self-specific automatic and involuntary attention allocation processing of the internal and external stimuli. In support, there was evidence that about 33% of the subjects reported that they heard the presentation of their own name but did not perceive other words repeatedly presented (Hiscock et al., 1999). Alexopoulos et al. (2012) found that self-name had a high cueing effect and had nothing to do with familiarity under either conscious or unconscious conditions and that it was difficult to suppress attention turning to self-name. Similar to auditory SRI processing, e.g., “cocktail party effect,” visual SRI also captured more attention and had high priority in recognition speed (Conway et al., 2001; Harris et al., 2004; Tacikowski and Nowicka, 2010). For example, using the self/other name judgment task with faces as distractors along with one’s own name, Bredart et al. (2006) found that the speed of people’s name judgment was significantly affected by their own faces. Moreover, it had been found that visual SRI as distractors had an interfering effect when it was unrelated to the task (Gronau et al., 2003; Harris and Pashler, 2004; Sui et al., 2012). In this study, we further investigated the processing of SRI using mismatch negativity (MMN) of event-related potentials (ERPs), a biomarker reflecting the preattentional change detection under non-attentional conditions (Frodl-Bauch et al., 1997; Näätänen, 2003).

Auditory MMN (aMMN) is a negative component elicited by occasional biased stimuli when subjects are in the repeated sequence of the same stimulus, with a latency of 150–250 ms, reflecting the individual’s automatic awareness of the changes of the current stimuli and the previous sequential stimuli (Näätänen et al., 1978). Similar to aMMN, converging evidence found the visual MMN (vMMN) related to automatic preattentional change detection of color (Czigler et al., 2002), shape (Grimm et al., 2009), size (Kimura et al., 2009), and orientation (Kimura et al., 2009). In addition, some complex visual information could also elicit vMMN (Berti and Schroger, 2001; Czigler et al., 2002). For example, several investigations found the significant bilateral occipital–temporal vMMN related to facial expressions, indicating that the facial expression content could be rapidly processed and stored in visual predictive memory representations under non-attentional conditions (Stefanics et al., 2012).

Previous studies have shown that, compared with irrelevant and relevant stimuli, SRI automatically captured attention during the preattentional stage and elicited MMN with a larger amplitude in the auditory channel (Tacikowski et al., 2011; Tateuchi et al., 2012; Graux et al., 2015). For example, Graux et al. (2013) found that self-sound elicited a larger MMN in the preattentional stage of auditory processing, which provided strong evidence for the automated processing in auditory channels. More specifically, it was evidenced that self-generated movement sounds, such as one’s own finger snapping sounds, could elicit MMN, indicating that preferential processing of the characteristic sensory features of SRI (self-related movement sounds) occurred prereflectively and without being aware of it (Justen and Herbert, 2016). Different from previous studies, Rachman et al. (2019) investigated whether the same expressive changes (pitch variations, inflections, and timbre) were processed differently on self-voice compared to a stranger’s voice and found that strangers’ voice deviants generated earlier MMN onset responses, indicating that expressive changes on a stranger’s voice were highly prioritized in auditory processing compared to identical changes on self-voice. Although the above findings are inconsistent, it is generally believed that auditory SRI could elicit MMN, reflecting the preattentional change detection processing of SRI.

Until now, there are many studies on MMN elicited by SRI, most of which focused on the auditory channel. By contrast, there are no experiments specifically to explore whether visual SRI could elicit MMN. In this study, we would explore the preattentive automatic processing mechanism of visual SRI by examining whether it could elicit vMMN. It has been shown that, as one of the form of visual information, self-related texts were highly efficient reading materials to study cognitive processing (Tanguay et al., 2022). Previous studies usually used personal names as experimental materials because names were very stable and difficult to change in most cases and had become a stable marker of personal identity (Tacikowski and Nowicka, 2010).

As a place of upbringing, birthplace is full of personal memories, which own similar features with faces, self-names, etc., and should have a certain degree of self-relevance. To date, there has been no research on SRI processing using city names as an experimental material. Therefore, we chose city names, including birthplace, with no more than two characters, that could reflect different levels of self-relevance without causing difficulty in the reading cognitive load as experimental materials to verify the automatic processing of SRI. If vMMN could be successfully elicited by self-related texts of an individual’s birthplace, it would provide further electrophysiological evidence for automatic processing of SRI and also make mutual confirmation with previous studies on aMMN elicited by SRI in auditory channels.

## Materials and Methods

### Participants

Thirty-five right-handed participants (5 females; 18–25 years old) were recruited from the Air Force Medical University. They had normal or corrected vision, a normal mental health status (referred to the mental records), no physical discomfort, and no history of neurological diseases and mental illness. This study was reviewed and approved by the Air Force Medical University, and all participants signed informed consent to participate in this study.

### Materials

The stimuli used in the study were city names (Chinese words of two characters). We conducted a preliminary interview on the consistency of the subjects’ past childhood experiences and the happiness of their native families to ensure that they have not changed their usual place of residence from birth to growing up and have a happy childhood. Then, each participant was asked to list 25 city names, including the birthplace, which were all double characters in Chinese. A total of 161 city names were obtained and the *City Relevance Questionnaire* was compiled. Each participant was asked to rate their relevance on a scale of 1 (very irrelevant) to 7 (remarkably relevant).

According to the purpose and the hypothesis of this experiment, 6 standard stimuli and 2 deviant stimuli were needed. One deviant stimulus was birthplace’s name and the other stimuli was the city names with relatively low self-relevance. Xi’an was the city where the participants’ university is located, so we chose Xi’an as another deviant stimulus. The standard stimuli were all the city names with lower self-relevance than the deviant stimuli, so we chose 6 cities totally unrelated to the participants as the standard stimulus. Therefore, in addition to each subject’s birthplace, 7 non-birthplace cities were selected, namely, Xi’an, Dongguan, Kashgar, Jinzhou, Wuwei, Zhoukou, and Qionghai. We controlled physical properties of stimuli, such as brightness, size, and the number of strokes, at the similar level, which were only different in meaning.

We adopted one-way ANOVA and the Bonferroni posttest method, which took the city names as an independent variable but degrees of self-relevance as a dependent variable. The three types of self-related cities had significant statistical differences [*F*(2,277) = 636.25, *p* < 0.001, partial η^2^ = 0.821]. As shown in Table 1, birthplace had the highest self-relevance, and there was a statistically significant difference in self-relevance with other cities (*p* < 0.001), which was defined as “high SRI.” The self-relevance of Xi’an was in medium and there was a statistically significant difference between the self-relevance of Xi’an and other cities (*p* < 0.001), which was defined as “medium SRI.” The other six cities had the lowest self-relevance, and there was no statistically significant difference in mutual self-relevance [*F*(5,204), *p* = 0.117, partial *η^2^* = 0.42], which was defined as “low SRI.”

**TABLE 1:** Assessment results of city self-relevance (*n* = 35, $\bar{\text{X}}\pm \text{SD}$).

Number	City name	Self-relevance	Classification
1	Birthplace	6.62 ± 0.50^ab^	High
2	Xi’an	4.61 ± 1.20^c^	Medium
3	Dongguan	1.77 ± 1.23	Low
4	Kashgar	1.43 ± 0.95	Low
5	Zhoukou	1.46 ± 1.01	Low
6	Jinzhou	1.60 ± 1.21	Low
7	Qionghai	1.40 ± 0.74	Low
8	Wuwei	1.40 ± 0.85	Low

*^a^Birthplace was compared with Xi’an, p < 0.001.*

*^b^Birthplace was compared with Dongguan, Kashgar, Jinzhou, Wuwei, Zhoukou, and Qionghai, p < 0.001.*

*^c^Xi’an was compared with Dongguan, Kashgar, Jinzhou, Wuwei, Zhoukou, and Qionghai, p < 0.001.*

All participants of the study were born in one of the 12 cities, including Rizhao, Jurong, Anyang, Zhenjiang, Jinan, Wuxi, Nanjing, Taizhou, Yancheng, Yan’an, Danyang, and Guang’an. Therefore, experimental materials for each participant were the same except for the birthplace’s name. For example, if participant A’s birthplace was Rizhao, the deviant stimuli were Rizhao and Xi’an and the standard stimuli were Dongguan, Kashgar, Jinzhou, Wuwei, Zhoukou, and Qionghai. If participant B’s birthplace was Jurong, the deviant stimuli were Jurong and Xi’an and the standard stimuli were Dongguan, Kashgar, Jinzhou, Wuwei, Zhoukou, and Qionghai.

### Procedure

This study adopted the visual Oddball paradigm, which took low self-related cities as the standard stimuli (360 trials with a presenting probability of 75%) and high and medium self-related cities as the deviant stimuli (60 trials with a presenting probability of 12.5%, respectively). The first 10 trials of the oddball sequence were the standard stimuli and no less than two standard stimuli were between the successive deviant stimuli.

**FIGURE 1:**
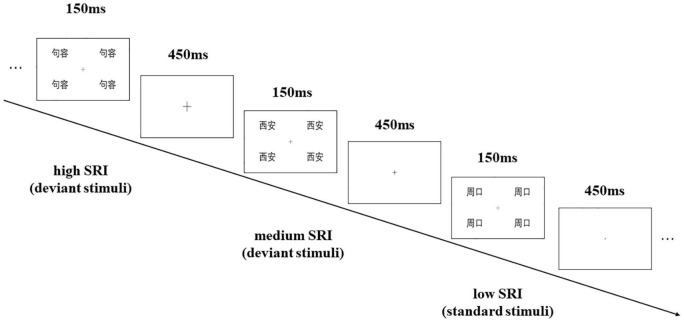
Sample of the visual mismatch negativity (vMMN) procedure with “Jurong” as the participant’s birthplace.

Each stimulus picture is composed of the four same city names located in the four quadrant corners of the screen (top left, top right, bottom left, and bottom right), with a presenting time of 150 ms. The interstimulus interval (ISI) between the two pictures was 450 ms. In the experiment, the participants were asked to sit in front of the computer screen at about 70 cm away, were required to pay attention to the “+” in the center of the visual field, and were asked to press the button (counterbalance among participants) as accurately as possible when the size of “+” changed. The experiment flow figure is shown in Figure 1. Before the formal experiment, the participants completed a practice sequence consisting of 24 trials, which were not used in the formal experiment.

### Electroencephalography Recording and Analysis

Electroencephalography signals were continually recorded (sampling rate of 1,000 Hz and bandwidth of 0.01–100 Hz) by the MitSar 202 EEG DC Acquisition System^1^ with a 32 channel Ag/AgCl electrode cap based on the international 10–20 electrode system. The reference electrode was placed at the tip of the nose. The vertical electro-oculographic (VEOG) electrode was placed 1 cm above and 1 cm below the right orbit, and the horizontal electro-oculographic (HEOG) electrode was placed 1 cm outside the right eye and 1 cm outside the left eye. The impedance of each electrode in the experiment was kept below 5 kΩ.

The EEG data were analyzed offline using WinEEG software (see text footnote 1). After removing the ocular artifacts by the ICA method, the EEG signals were segmented from 100 ms before the stimulus onset to 600 ms after the poststimulus onset. Segments with erroneous reactions and with voltages exceeding ±100 μV were removed. The average ERPs waveform of each participant was filtered by a phase-shift-free low-pass filter of 30 Hz (24 dB/Oct). Each type of stimuli included 40 trials at least.

**FIGURE 2:**
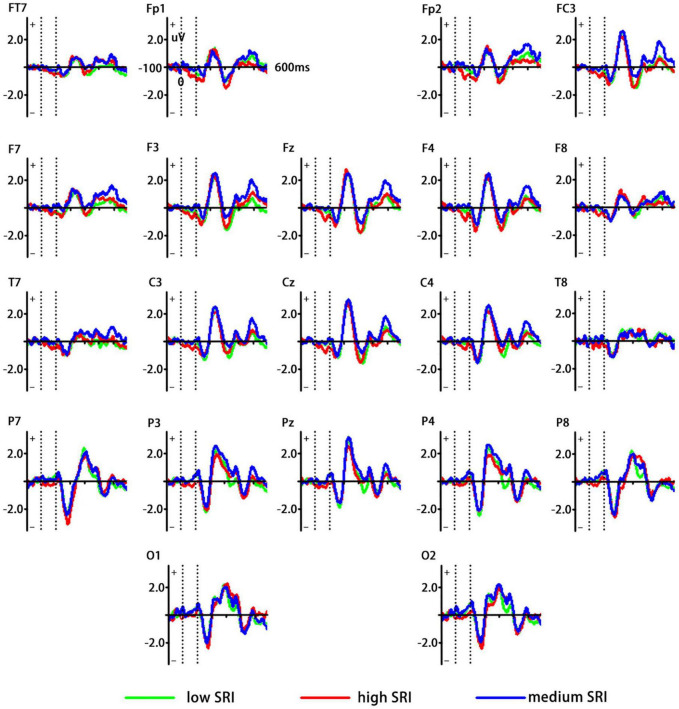
Grand averaged event-related potentials (ERPs) waveforms elicited by the standard [low self-related information (SRI)] and the deviant (high and medium SRI) stimuli, respectively. *Green curve* represents low SRI; *red curve* represents high SRI; and *blue curve* represents medium SRI.

Generally, vMMN was obtained by subtracting ERPs elicited by the standard stimuli from ERPs by the deviant stimuli. Figure 2 shows different waveforms between ERPs related to high and medium SRI (deviant stimuli) and low SRI (standard stimuli). Based on the grand-averaged waveforms and previous vMMN studies, the mean amplitudes at the occipital–temporal sites (P3, P4, P7, P8, O1, and O2) were measured between 180 and 300 ms of the poststimulus onset.

### Statistical Analysis

Statistical product and service solutions (SPSS) version 19.0 was used for statistical analysis, and all the data were tested for normal distribution and homogeneity of variance. The *t*-test and repeated measures ANOVA were used in this study, with *p* < 0.05 as the statistical significance level. For each time window, i.e., 180–220 ms, 220–260 ms, and 260–300 ms based on the continuous interval measurement method, the three-way repeated measurement ANOVA was conducted with a relevance degree (high and medium self-relevance), brain region (left and right), and sites (P3/4, P7/8, O1/2) as within-subject factors. In addition, it was necessary to verify the existence of vMMN by the *t*-test between the average amplitude of vMMN and zero, regardless of high or low SRI.

### Cortical Source Analysis

Extrapolating the cortical origin of the ERPs effect (inverse computation) provided some useful hypotheses for the brain’s involvement in the respective cognitive processes. In this study, we used standardized low-resolution electromagnetic tomography (sLORETA) to analyze cortical sources of vMMN associated with automatic processing of high SRI. The sLORETA method used a standardized Boundary Element Method (BEM) volume conduction model and extended electrode coordinates [Montreal Neurological Institute (MNI) stereotaxic coordinates] based on an effective head surface positioning system to obtain a standard current density image with zero localization error (Bhagwat et al., 2021). It employs the current density estimate given by the minimum norm solution, and localization inference is rooted on standardized values of the current density estimates and, thus, sLORETA is capable of exact (zero-error) localization (Pascual-Marqui, 2002).

## Results

**FIGURE 3:**
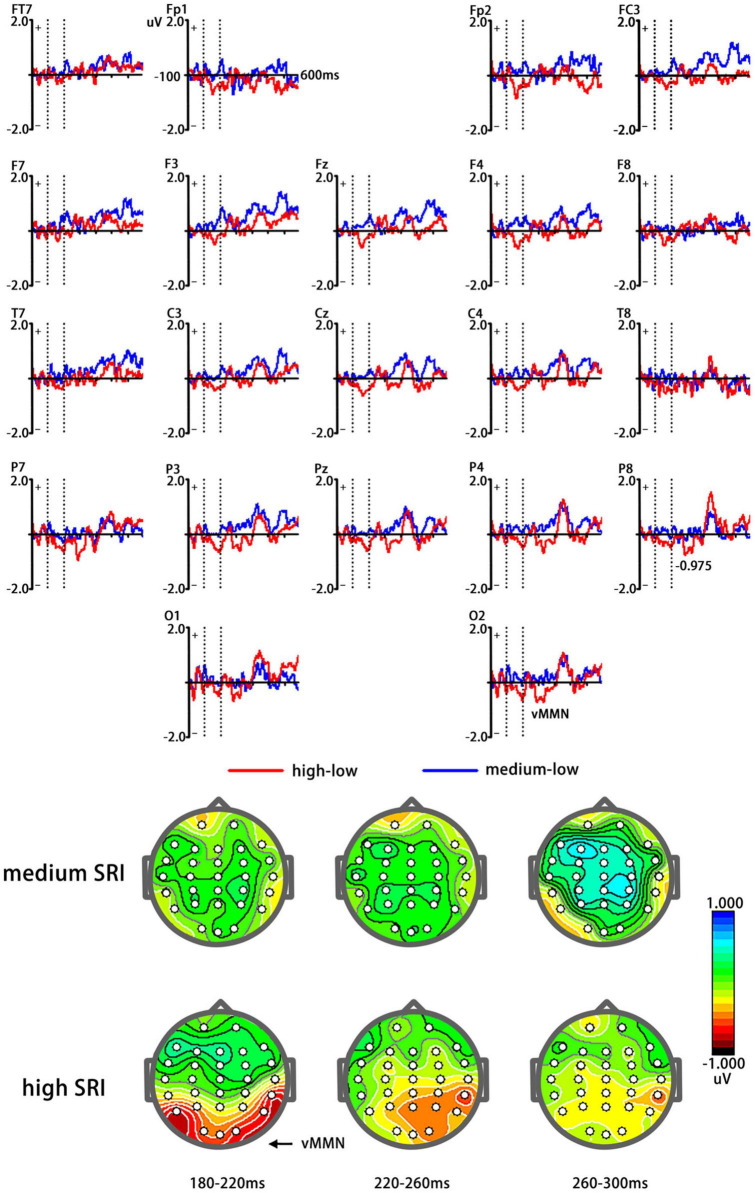
Visual mismatch negativity elicited by high (birthplace) and medium (current city) SRI, respectively [**(A)** *top*]. *Red curve* represents vMMN elicited by high SRI, whereas *blue curve* represents vMMN elicited by medium SRI. A topographic map of vMMN elicited by high (birthplace) and medium (current city) SRI [**(B)** *bottom*].

Figure 2 shows different waveforms between ERPs related to high and medium SRI (deviant stimuli) and low SRI (standard stimuli). Figure 3 shows vMMN as well as topographic maps elicited by high and medium SRI, respectively. The ANOVAs for mean amplitudes of vMMN were conducted to three time windows, respectively.

### 180–220 ms

The average amplitudes of the vMMN elicited by high SRI (−0.76 μV) were significantly larger (more negative) than that by medium SRI [0.08 μV; *F*(1,24) = 5.51, *p* = 0.026]. The main effect of the electrode site was significant [*F*(2,48) = 6.84, *p* = 0.004], showing a maximum of −0.975 μV at the P7/8 site. Other effects were not significant (*ps* > 0.1).

The *t*-test between the average amplitudes of vMMN and zero revealed that vMMN amplitudes were significantly lower than zero for high SRI (−0.95 μV, *t* = −3.10, *p* < 0.01, and −0.86 μV, *t* = −2.78, *p* = 0.01, for the left and the right occipital-temporal sites, respectively) but not for medium SRI (−0.01 μV and 0.01 μV for the left and the right occipital-temporal sites, respectively; *ps* > 0.1).

### 220–260 ms

Similar to the analysis of 180–220 ms, the mean amplitudes of vMMN elicited by high SRI (−0.79 μV) were significantly larger (more negative) than that by medium SRI [0.11 μV; *F*(1,24) = 4.47, *p* = 0.043]. Other main effects and interaction effects were not significant (*ps* > 0.1).

The *t*-test between the average amplitudes of vMMN and zero revealed that vMMN amplitudes were significantly lower than zero for high SRI (−0.83 μV, *t* = −2.43, *p* = 0.022, and −0.89 μV, *t* = −2.29, *p* = 0.029, for the left and the right occipital–temporal sites, respectively) but not for medium SRI (0.05 and 0.02 μV for the left and the right occipital-temporal sites, respectively; *ps* > 0.1).

### 260–300 ms

Neither the main effects nor the interactions were significant (*ps* > 0.1).

### Source Analysis

**FIGURE 4:**
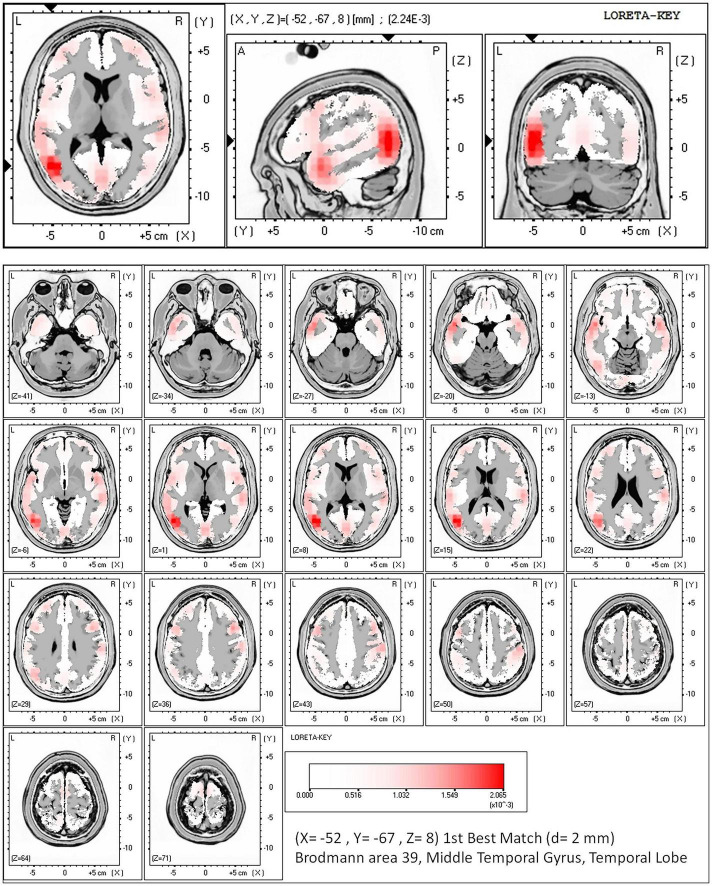
Cortical source analysis of vMMN elicited by high SRI (birthplace).

Source analysis was conducted for vMMN elicited by high SRI. The results showed that vMMN involved several brain regions mainly in the left occipital–temporal region, including the middle temporal gyrus (BA 39, *x* = −52, *y* = −67, *z* = 8; BA 21, *x* = −52, *y* = 3, *z* = −20; BA 22, *x* = −59, *y* = −46, *z* = 1), the posterior central gyrus (BA 40, *x* = −59, *y* = −25, *z* = 15), and the cuneus (BA 17, *x* = 4, *y* = −46, *z* = 1). The maximum cortical current density was found in the left middle temporal gyrus (2.59 E-3/cm^3^; BA 39, *x* = −52, *y* = −67, *z* = 8), as shown in Figure 4.

## Discussion

To explore automatic processing of visual SRI, we investigated whether birthplace as the SRI could elicit vMMN in the visual channel. Compared with low self-related city names, birthplace as the high visual SRI induced clear vMMN over the occipital–temporal brain region, whereas medium self-related city names did not elicit vMMN.

This study provided strong evidence that the birthplace elicited evident MMN vs. other cities, indicating that the birthplace is also as a specific SRI like own’ names and faces. As for the experimental materials in this study, most people visited many cities in their lifetime, but different cities have different meanings for them, such as work, study, living, business, and travel. There are also some cities they had heard about on the news, streaming media, and online but never visited them. On the one hand, the experimental materials we used in this study were all printed words. Even though there were three kinds of stimuli, they were all in Chinese characters. We had controlled their physical properties, such as brightness, size, and the number of strokes, at a similar level, which were only different in meaning. On the other hand, it is worth mentioning that hometown had a unique meaning for everyone, such as familiar relatives, kind teachers, amiable friends, and visited and played gardens, and constitute a schema that is deeply rooted in our hearts. Therefore, in this study, we divided cities with different degrees of self-relevance into high, medium, and low SRI, based on the results of the *City Relevance Questionnaire*, which specifically measures the degrees of relevance of different cities for each participant. We made personalized experimental materials according to the specific situation of each subject. Birthplace was regarded as high SRI. The place where participants were working or studying was regarded as medium SRI, while other places totally unrelated to individuals were regarded as low SRI. The results showed that, compared with low SRI as the standard stimuli, high SRI as the deviant stimuli could elicit significant vMMN, while medium SRI as the deviant stimuli did not elicit significant vMMN, suggesting that birthplace as high SRI was effectively processed by automatic preattention based on memory comparison in the visual channel. It might be that the stimuli involving SRI were evolutionarily important (Gray et al., 2004) and, therefore, automatically attracted attention. Compared with medium self-relevance cities, birthplace was a city with high self-relevance, which contained information pointing to self-concept and would cause automatic processing of attention. Therefore, it could be accepted that birthplace, also known as hometown, was an irreplaceable place in individuals’ hearts, which had internalized into a highly self-related and an inseparable part of the ego. This study provided objective evidence for the extension of self-concept, that is, birthplace was a part of the ego as high SRI. The role of hometown in self-construal went far beyond the place of work and study.

Previous studies on vMMN mainly focused on the change detection of visual simple features, such as color, shape, size, and orientation (Czigler et al., 2002; Grimm et al., 2009; Kimura et al., 2009), as well as some complex visual information, such as faces and visual printed words (Berti and Schroger, 2001; Czigler et al., 2002; Zhao and Li, 2006). In this study, we first found that high visual SRI could elicit an evident vMMN with the occipital–temporal scalp distribution and could extend the previous vMMN research. Moreover, aMMN has been used as a sensitivity indicator of automatically processing SRI. Compared with irrelevant and relevant information, SRI (e.g., self-voice and self-name) automatically captured attention in the preattentional stage and elicited a larger amplitude of MMN (Tacikowski et al., 2011; Tateuchi et al., 2012; Graux et al., 2015). For example, Holeckova et al. (2006) found that the participant’s own name generated more robust responses related to involuntary attention switching reflected by MMN and novelty P3 than a non-vocal stimulus, indicating that hearing one’s own first name automatically elicited a robust electrophysiological response (e.g., MMN), even in conditions of reduced consciousness like sleep. Similarly, Graux et al. (2013) examined the neural processes underlying own voice discrimination and found that the own voice discriminative response was associated with an early pre-MMN response involving a left inferior frontal component, the activity of which lasted throughout the time course of the discriminative response. Additionally, there was evidence that personal ringtone could elicit aMMN and P3a, indicating that the personal significance of mobile phones prompts the formation of individual memory representations (Roye et al., 2007, 2010). To date, it was generally accepted that people could automatically process the auditory SRI as revealed by MMN. The present findings extended the aMMN research of SRI, further indicating the automated processing of SRI, regardless of visual or auditory modality.

Interestingly, the cortical source analysis showed that vMMN elicited by high visual SRI was mainly located in the left occipital-temporal region. Previous studies showed that the vMMN were all distributed in the occipital–temporal lobe (Gayle et al., 2012), which was consistent with this study. Furthermore, the memory trace hypothesis based on sensory memory indicated that a memory trace would be formed in the brain when a stimulus acted on the body, and the memory traces of different types of stimuli were different (Naatanen et al., 2007). Evidence from a previous study has shown that the processing of writing materials occurred mainly in the left temporal occipital area (Wei et al., 2018), while the processing of faces occurred in the right temporal occipital area (Vogel et al., 2015). In this study, city names were used as the test stimuli, and the self-related vMMN elicited by them was mainly located in the left occipital–temporal region, which supported the memory trace hypothesis. Furthermore, Stefanics et al. used the “reverse block” design to support the approach-withdraw hypothesis, i.e., the left brain specifically processed positive/proximity emotions, while the right brain was dominant in processing negative/avoidant emotions (Alves et al., 2008). In this study, the processed cognitive materials we used were the birthplace texts materials, which could account for the results that vMMN elicited by birthplace was mainly located in that left occipital–temporal region in line with the left predominance of processing texts materials.

There are several limitations to this study. On the one hand, most of the participants were male. A large number of studies have shown that male and female participants had different cognitive characteristics (Alves et al., 2008). For instance, female participants had a greater advantage in terms of emotion perception (Li et al., 2008). On the other hand, cognitive/cold and emotional/hot dimensions (i.e., official relevance as a forever part of an official record vs. city of the first kiss) may play a role in the construction of the self-related cities, which need further study. Additionally, a further comparison could be made among birthplace and sound, name, facial expressions, etc., as the stimulus material. Therefore, it was necessary to further compare the differences between different stimulus materials.

## Conclusion

In conclusion, to explore automatic processing of SRI, we investigated whether birthplace as the high SRI could elicit vMMN in the visual channel. We found that vMMN could be elicited by high self-related city information but not by medium SRI with an occipital–temporal scalp distribution, indicating that, under the non-attentional condition, compared with low SRI, only high SRI could be effectively processed automatically in the preattentional stage in the visual channel and provides further electrophysiological evidence for automatically processing SRI. In future, we will further combine EEG, eye movement, functional MRI (fMRI), and other technologies to reveal the mechanism underlying the automatic processing of SRI from the time-spatial multimodality perspective.

## Data Availability Statement

The original contributions presented in the study are included in the article/supplementary material, further inquiries can be directed to the corresponding author/s.

## Ethics Statement

The studies involving human participants were reviewed and approved by the Air Force Medical University. The patients/participants provided their written informed consent to participate in this study.

## Author Contributions

SC, XiL, and QZ wrote the manuscript. YW, YG, WH, and YC contributed to the data collection. TF, HW, SW, and FA contributed to the data analysis. XW, LZ, and XuL designed and revised the manuscript. All authors contributed to the article and approved the submitted version.

## Conflict of Interest

The authors declare that the research was conducted in the absence of any commercial or financial relationships that could be construed as a potential conflict of interest.

## Publisher’s Note

All claims expressed in this article are solely those of the authors and do not necessarily represent those of their affiliated organizations, or those of the publisher, the editors and the reviewers. Any product that may be evaluated in this article, or claim that may be made by its manufacturer, is not guaranteed or endorsed by the publisher.

## Footnotes

1
www.mitsar-eeg.com
